# Novel Methinic Functionalized and Dendritic C-Scorpionates

**DOI:** 10.3390/molecules23123066

**Published:** 2018-11-23

**Authors:** Luísa M. D. R. S. Martins, Riccardo Wanke, Telma F. S. Silva, Armando J. L. Pombeiro, Paul Servin, Régis Laurent, Anne-Marie Caminade

**Affiliations:** 1 Centro de Química Estrutural, Instituto Superior Técnico, Universidade de Lisboa, Av. Rovisco Pais, 1049-001 Lisboa, Portugal; rwanke@tecnico.ulisboa.pt (R.W.); tsilva@dem.isel.ipl.pt (T.F.S.S.); pombeiro@tecnico.ulisboa.pt (A.J.L.P.); 2 Laboratoire de Chimie de Coordination du CNRS, 205 route de Narbonne, BP 44099, F-31077 Toulouse CEDEX 4, France; paul.servin@xeroscleaning.com (P.S.); regis.laurent@lcc-toulouse.fr (R.L.); anne-marie.caminade@lcc-toulouse.fr (A.-M.C.); 3 LCC-CNRS, Université de Toulouse, CNRS, F-31077 Toulouse, France

**Keywords:** C-scorpionate, pyrazolyl, tris(pyrazolyl)methane, functionalization, coordination flexibility, dendrimer, catalyst, C-C coupling, Sonogashira, Heck

## Abstract

The study of chelating ligands is undoubtedly one of the most significant fields of research in chemistry. The present work is directed to the synthesis of new functionalized derivatives of tripodal C-scorpionate compounds. Tris-2,2,2-(1-pyrazolyl)ethanol, HOCH_2_C(pz)_3_ (**1**), one of the most important derivatives of hydrotris(pyrazolyl)methane, was used as a building block for the synthesis of new functionalized C-scorpionates, aiming to expand the scope of this unexplored class of compounds. The first dendritic C-scorpionate was successfully prepared and used in the important industrial catalytic reactions, Sonogashira and Heck C-C cross-couplings.

## 1. Introduction

**Figure 1:**
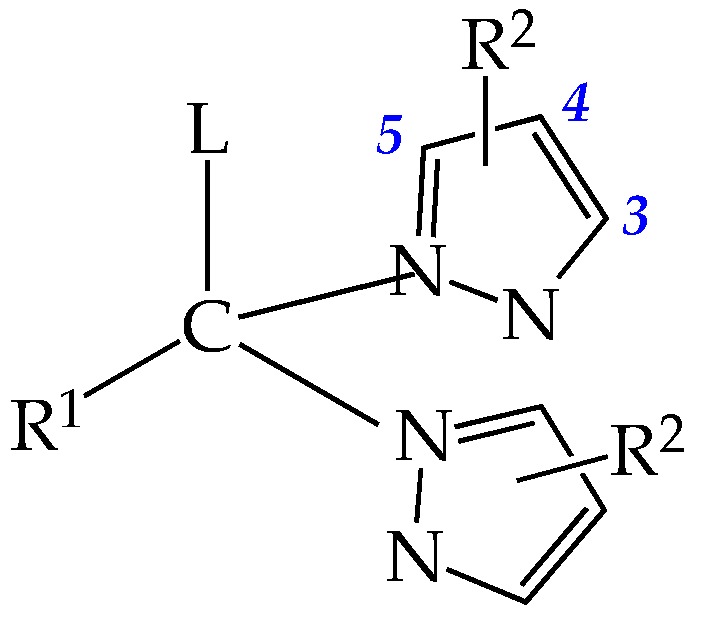
General C-scorpionate structure: homoscorpionate (L = pyrazolyl) or heteroscorpionate (L ≠ pyrazolyl).

Tripodal scorpionates are a relevant example of chelating ligands and hold a primary role in many areas of chemistry [1,2,3,4,5,6,7,8,9,10,11]. They consist of pyrazole-based compounds, known as poly(pyrazolyl) ligands, formed from at least two N-deprotonated pyrazole rings bound to a main group atom (e.g., B or C) through one nitrogen atom of the pyrazolyl ring (Figure 1).

Their designation, scorpionates, in particular of tris(pyrazolyl) ligands, arises from their peculiar coordination mode: with the three nitrogen atoms of the corresponding pyrazolyl rings able to occupy three facially adjacent vacant positions of the coordination sphere of a metal center. This configuration (i.e., *fac*-chelation) mimics the analogue position of the scorpion that uses three weapons (two claws and one poisonous sting) to attack its prey.

A similar analogy has been described for another class of chelating ligands, the pincer ligands. The latter are, in fact, characterized by their peculiarity to bind two or three adjacent coplanar positions of the coordination sphere of a metal center. Differently, the scorpionate ligands extend their coordination ability out of a planar dimension, employing the third ‘arm’ to a facially capping coordination.

The boron derivatives tris(pyrazolyl)borates [2,3,4] have become one of the most highly studied class of ligands [2,3,4,5,6,7,8,9,10,11,12,13] being used to synthesize complexes of most metals of the periodic table. Their carbon analogues, tris(pyrazolyl)methanes, maintain the tripodal face capping feature and the electron donor ability, but differ from the formers in the holding charge. Thus, tris(pyrazolyl)methanes are able to coordinate to metal centers in a *N*,*N*,*L*-chelating mode (L = N or other donor atom) or could afford different coordination modes (e.g., κ^2^-mode, i.e., bidentate, *N*,*N*- or *N*,*L*-, see Figure 1), adapting their geometry to the electronic demands of the metal center, or even acting as bridging ligands [6,13].

The complicated and time-consuming synthesis of tris(pyrazolyl)methanes in comparison to that of borate analogues is the main reason for the underdevelopment of such a class of ligands and has encouraged us in exploring their functionalization.

Several positions of the scorpionate molecule are recognized [2,6,13] to have a great significance for functionalization. The two main sites of functionalization are: (i) the apical position (R^1^, Figure 1) and (ii) the heterocyclic ring (R^2^, Figure 1). Through the modification of these sites [(i), (ii) or (i) and (ii)] it is possible to achieve unique effects on the coordination behavior of the corresponding ligand.

It is expected that ring substituents would have an effect on the steric and electronic donor/acceptor properties of the ligand. It is possible to define separately the effect of the substituents at the pyrazolyl ring: the major contribution of the substituents at positions 3 and 5 (see Figure 1) is of steric nature. For example, groups at position 3 are facially oriented toward the coordination to the metal center and hold an important role for the modulation of tris(pyrazolyl)methane coordination chemistry [14]. In the case of bulky substituents (e.g., *i*-Pr, Ph or *t*-Bu), the ligand is forced to engage a definite coordination geometry. In particular, for those metals with a high affinity for the octahedral *N*_6_-coordination mode, e.g., Fe(II), this steric limitation obliges them to abandon the typical full-sandwich coordination. Moreover, it has been reported [15] that substituents at position 3 could be directly involved in the coordination to the metal center. Furthermore, it has been proved that the bulky substituents at the 3-position could also enhance the thermodynamic and hydrolytic stability of the corresponding complexes.

On the other hand, it has been recognized that synthetic progress toward the functionalization of the central methine carbon atom of tris(pyrazolyl)methanes would influence the properties of their complexes, as well as allow their attachment on a solid support, what can be of great significance for catalytic applications of C-scorpionates toward new fields [6,7,8,16,17,18,19,20,21,22,23,24]. In spite of the electron withdrawing effect of the three pyrazolyl rings of hydrotris(pyrazolyl)methane compound, the central methine (tertiary) carbon is stable. Therefore, the substitution at this carbon atom can be achieved by reacting a suitable electrophile with the carbanion [C(pz)_3_]^−^ formed by deprotonation of hydrotris(pyrazolyl)methane [25].

An interesting functionalization approach, particularly for researchers dealing with catalysts heterogenization [17,18,19,20,21,22,23,24,26,27,28,29], would be the possibility of modifying C-scorpionates properties, prompting them to bind to a dendritic support. Dendrimers, as hyperbranched macromolecules (synthesized from a central core using repetitive branching elements) with perfectly defined multifunctionalized structures, are particularly suitable for catalytic applications. In fact, the presence of easily accessible multiple terminal functions enables their use in efficient homogeneous catalytic processes [30,31].

One of the most important derivatives of hydrotris(pyrazolyl)methane for the development of a synthetic plan for further functionalization is the hydroxy-methylene tris-2,2,2-(1-pyrazolyl)ethanol, HOCH_2_C(pz)_3_ (**1**), (Figure 1, R^1^ = pyrazolyl, L = CH_2_OH), prepared by a mild deprotonation of the apical methine carbon of hydrotris(pyrazolyl)methane and successive nucleophilic attack to paraformaldehyde [32]. The alkoxide could react with a large variety of species that have inspired this work.

Herein, HOCH_2_C(pz)_3_ (**1**) has been used as a building block for the synthesis of new tris(pyrazolyl)methane type C-scorpionates, aiming at to expand the scope of this unexplored class of compounds, namely e.g., modifying their properties by grafting them on a dendrimer.

Moreover, the successfully prepared dendritic scorpionate, the first of its class, was used, as well as its scorpionate precursor (**1**), for the in situ formation of a Pd-catalyst for C-C bond-forming reactions. As test reactions, the catalytic Sonogashira and Heck C-C cross-couplings were chosen. In fact, the Sonogashira reaction is the most useful method to prepare conjugated enynes and arenynes by coupling of sp^2^-hybridized carbons and terminal alkynes [33,34,35] resulting in important intermediates for the synthesis of pharmaceuticals. In addition, the versatile coupling of organic halides with olefins in the presence of palladium and base, the Heck reaction, is widely applied for the synthesis of pharmaceuticals [36,37] and for other industrial applications.

To our knowledge, this is the first time that the successful use of a dendritic C-scorpionate for C-C coupling reactions is reported.

## 2. Results

Tris-2,2,2-(1-pyrazolyl)ethanol, HOCH_2_C(pz)_3_ (**1**), was used as a “chemical scaffold” for the development of synthetic plans for further functionalization of hydrotris(pyrazolyl)methane.

### 2.1. Functionalization at the Methinic Carbon

**Figure 2:**
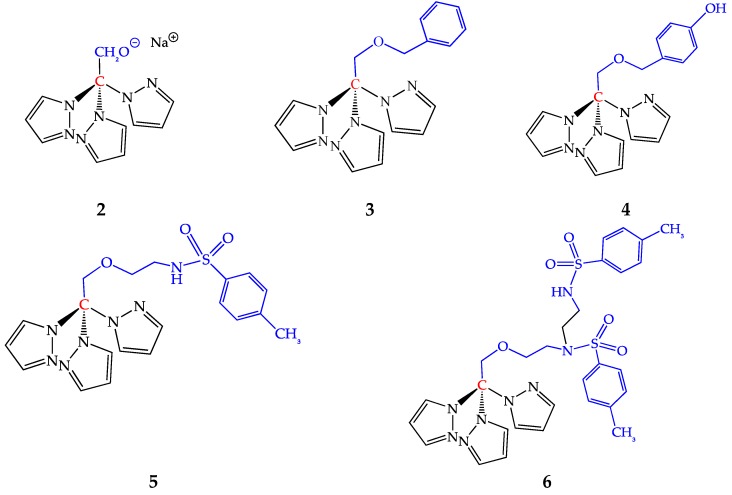
Novel functionalized tris(pyrazolyl)methane type C-scorpionates.

The novel C-scorpionates prepared in this work from tris-2,2,2-(1-pyrazolyl)ethanol are presented at Figure 2 and their synthesis is described below.

**Scheme 1:**
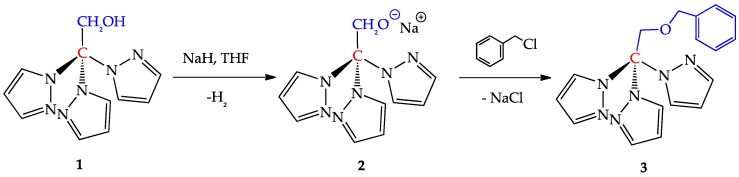
Sodium tris-2,2,2-(pyrazol-1-yl)ethanoate, NaOCH_2_C(pz)_3_ (**2**), and tris-2,2,2-(pyrazol-1-yl)ethoxy)benzyl, PhCH_2_OCH_2_C(pz)_3_ (**3**), obtained from tris-2,2,2-(1-pyrazolyl)ethanol HOCH_2_C(pz)_3_ (**1**).

The hydroxy group of tris-2,2,2-(1-pyrazolyl)ethanol, HOCH_2_C(pz)_3_ (**1**), was easily deprotonated with sodium hydride (1 eq.), in a classical procedure of deprotonation of alkyl alcohols [38]. Its sodium salt (sodium tris-2,2,2-(pyrazol-1-yl)ethanoate, NaOCH_2_C(pz)_3_ (**2**), Scheme 1) is usually used instantaneously for the next step with a suitable electrophile to give the desired product. However, we were able to isolate the alkoxide, after evaporation of the solvent (tetrahydrofuran, THF) and crushing the residue in dry pentane. The pale-yellow solid (**2**) could be stored under dinitrogen at room temperature, maintaining its stability, for several days.

The reactivity of sodium alkoxide of tris-2,2,2-(1-pyrazolyl)ethanol (**2**) has been studied with benzyl chloride (Scheme 1). The O-benzylated product, tris-2,2,2-(pyrazol-1-yl)ethoxy)benzyl, PhCH_2_OCH_2_C(pz)_3_ (**3**, pz = pyrazolyl) has been isolated after chromatographic purification in a reasonable yield.

In fact, the alkoxide group of (**2**) exhibited higher reactivity than the carbanion derived from deprotonation of hydrotris(pyrazolyl)methane, which reaction with benzyl chloride did not proceed.

**Scheme 2:**
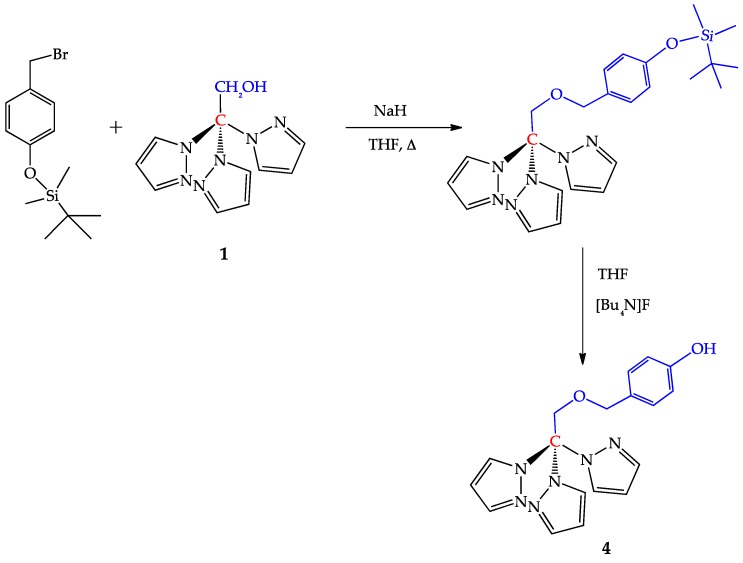
The phenolic C-scorpionate (4-(2,2,2-tris(1-pyrazolyl)ethoxymethyl)phenol, 4-OH-C_6_H_4_CH_2_OCH_2_C(pz)_3_, (**4**) obtained from reaction of tris-2,2,2-(1-pyrazolyl)ethanol (**1**) and tert-butyl dimethyl silyl protected 4-(bromomethyl)phenol.

The related 4-(2,2,2-tris(1-pyrazolyl)ethoxymethyl)phenol, 4-OH-C_6_H_4_CH_2_OCH_2_C(pz)_3_, (**4**) was achieved from the reaction of tris-2,2,2-(1-pyrazolyl)ethanol (**1**) and a *tert*-butyl dimethyl silyl protected 4-(bromomethyl)phenol (Scheme 2). The first step was the protection of the phenol of 4-hydroxybenzaldehyde by reaction with *tert*-butyldimethylsilane chloride and imidazole in THF (see Scheme S1 and NMR characterization of involved species, Supplementary Information (SI)). Then, the aldehyde group was reduced to alcohol with a retained protection on the phenol function. A better leaving group, bromide, replaced the alcohol, through two consecutive substitution reactions (See Scheme S1, SI), leading to the desired *tert*-butyl dimethyl silyl protected 4-(bromomethyl)phenol. Its reaction with a slurry of NaH and tris-2,2,2-(1-pyrazolyl)ethanol in THF yielded the *tert*-butyl dimethyl silyl protected (tris-2,2,2-(pyrazol-1-yl)ethoxy)-4-phenol, which undergone deprotection by solubilization in THF at low temperature and addition of tetrabutylammonium fluoride (Scheme 2).

**Scheme 3:**
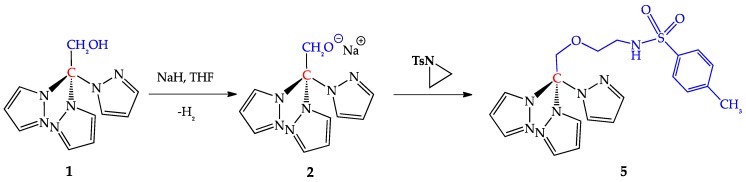
Synthesis of N-tosyl-2-(tris-2,2,2-(1-pyrazolyl)ethoxy)ethaneamine, TsNHCH_2_CH_2_OCH_2_C(pz)_3_ (**5**) (Ts = tosyl, pz = pyrazolyl) from tris-2,2,2-(1-pyrazolyl)ethanol (**1**).

Another type of functionalization of tris-2,2,2-(1-pyrazolyl)ethanol (**1**) has been developed in order to connect an NHR moiety to the scorpionate scaffold (Scheme 3) that holds important features in terms of hydrosolubility (i.e., the protonable group increases drastically the hydrophilicity of the compound), coordination chemistry and further functionalization (e.g., reaction with electrophiles or N-alkylation).

The alkylation of the hydroxy group of (**1**) with tosylated-aziridine could provide a derivative of hydrotris(pyrazolyl)methane bearing a longer alkyl chain with amino termination. Therefore, sodium ethanoate (**2**) was reacted with tosylated aziridine to yield quantitatively TsNHCH_2_CH_2_OCH_2_C(pz)_3_ (**5**) (Ts = para-toluenesulfonyl, tosyl, Scheme 3). Tosyl-protected azidirine was prepared according to the known procedure [39,40]: complete tosylation of ethanolamine, with subsequent cyclization upon deprotonation of sulfonamide with potassium hydroxide (see Scheme S2, SI). Compound (**5**) is stable in air, while its crystallization remained difficult due to the presence of the additional sulfonamido alkyl chain.

It was found that a second substitution at the amine N atom occurs during the formation of (**5**), leading to TsNHCH_2_CH_2_TsNCH_2_CH_2_OCH_2_C(pz)_3_ (**6**) (Figure 2) which was isolated (23% yield) from chromatographic purification of (**5**). The reaction can be tuned towards higher yields of (**5**) or (**6**) (see Experimental).

Unfortunately, the several attempts (different approaches, see Scheme S3, SI) to obtain the primary amine, by removing the tosyl protection, led to decomposition of the tris(pyrazolyl) backbone. In fact, most methods [41] to remove the N-protection involve extremely acidic conditions that promote the degradation of the C-scorpionate compound.

Moreover, different synthetic pathways have been attempted, without success, to replace the -OH moiety by the amino group. The activation of the -OH group to generate an efficient leaving group has been performed: mesylation or tosylation of tris-2,2,2-(1-pyrazolyl)ethanol yields easily the desired product. The latter, surprisingly, appears highly stable and every attempt to replace the mesylate (or tosylate) group failed. Harsh reaction conditions (i.e., NaI excess, DMF, 3 days, reflux) leave the compound unchanged with traces of the desired product. The unexpected stability of the mesylate derivative of tris-2,2,2-(1-pyrazolyl)ethanol could be partially explained considering the steric hindrance of the three pyrazolyl rings around the methylene moiety, they can obstruct the access to substitution that should occur via a SN2 mechanism.

Efforts to replace the hydroxy group by a halogen were also unsuccessful: simple chlorination, in mild conditions (e.g., oxalyl chloride, dichloromethane, room temperature) or in drastic conditions (e.g., thionyl chloride, neat, reflux) did not occur. Similarly, bromination (via phosphorus tribromide) led to the decomposition of the scorpionate scaffold to restore free pyrazole. All processes act in strong acidic range of pH (HCl or HBr is formed) and this could lead to a partial decomposition of the C-scorpionate.

To overcome the above difficulties, the Mitsunobu/Gabriel synthesis [42,43] has been carried out (see Scheme S4, SI). The substitution of hydroxyl group for a primary amine (i.e., -NH_2_) was accomplished although the final yield of the amino derivative was not satisfactory.

Different pathways to prepare the carboxylic acid derivative of hydrotris(pyrazolyl)methane have been attempted (see Scheme S5, SI). The carboxylic acid functionalization of the methine carbon atom is expected to be of great significance as a pro-ligand, due to its unique properties of solubility (hydrophylicity) and coordination ability, taking into account the known [44,45] coordination chemistry of its analogue bis(pyrazolyl)acetate. The apical proton of hydrotris(pyrazolyl)methane has been successfully removed and the carbanion carboxylated to obtain the lithium carboxylate Li[OOC-C(pz)_3_], characteristic for its νCO_2_ bands at 1704 and 1682 cm^−1^. This derivative has also been achieved by nucleophilic substitution of trichloroacetic acid or by oxidation of the terminal alcohol tris-2,2,2-(1-pyrazolyl)ethanol. However, at the final stage, the addition of acid to the carboxylate solution results in the decarboxylation of the derivative and formation of the starting hydrotris(pyrazolyl)methane. Steric and electronic effects could induce the high instability of the corresponding carboxylic acid. A proposed decarboxylation mechanism assisted from the nitrogen atom of an adjacent pyrazolyl ring is shown in Scheme S5 of SI.

### 2.2. Dendritic Functionalization

**Scheme 4:**
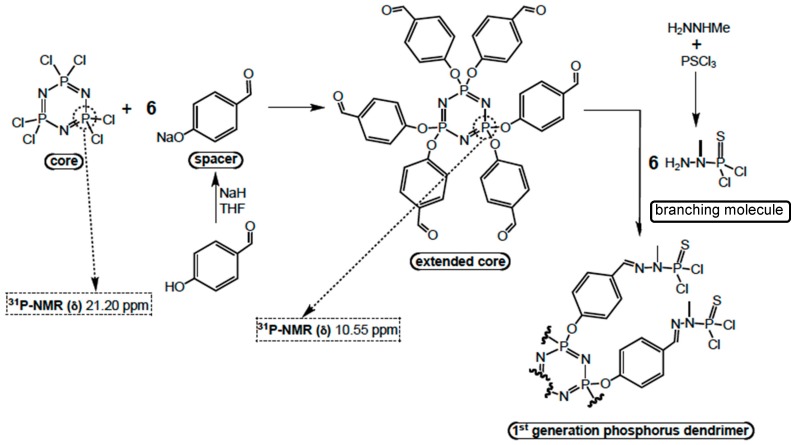
Synthesis of the first generation of the phosphorus dendrimer (N_3_P_3_-G_c1_).

The possibility to support tris-2,2,2-(1-pyrazolyl)ethanol (**1**) on a dendritic surface has been explored. The class of dendrimers used for this purpose has been the N_3_P_3_-type, known as phosphorus dendrimer [31,46]. In general, this dendrimer is initiated by the phosphonitrilic chloride trimer (N_3_P_3_Cl_6_, Scheme 4). From this central core, using a “spacer” and a “branching molecule” (see Scheme 4), a first-generation dendrimer suitable for our purposes has been prepared [47,48]:

- the nucleophilic substitution of the core N_3_P_3_Cl_6_ with 6 equivalents of previously prepared sodium salt of 4-hydroxybenzaldehyde (spacer), led to a sort of “extended core”. This product is not yet a dendrimer, since the branching points were not multiplied but stayed the same;
- the “branching molecule” was obtained from monomethylhydrazine with thiophosphonylchloride (solubilized in chloroform);
- six equivalents of *N*-methylhydrazido thiophosphonyldichloride are condensed with the aldehydic terminations of the macromolecule to yield the first-generation phosphorus dendrimer.

The above multistep synthesis has been monitored by NMR. The first step, that leads to the macromolecule bearing six-aldehydic terminations, proceeded overnight in THF for completion until the complete disappearance of phosphorus signal at δ 21.20 ppm in the ^31^P-{^1^H}-NMR spectrum (corresponding to phosphorus atoms in the starting material N_3_P_3_Cl_6_) and the presence of higher shielded resonance, at δ 10.55 ppm, ascribed to the product (Scheme 4) was observed. Successively, the ^1^H-NMR experiments allowed to confirm the formation of the final product: the aldehyde and the hydrazone protons, of the intermediate and final product were detected at δ 9.89 and 7.63 ppm, respectively.

**Scheme 5:**
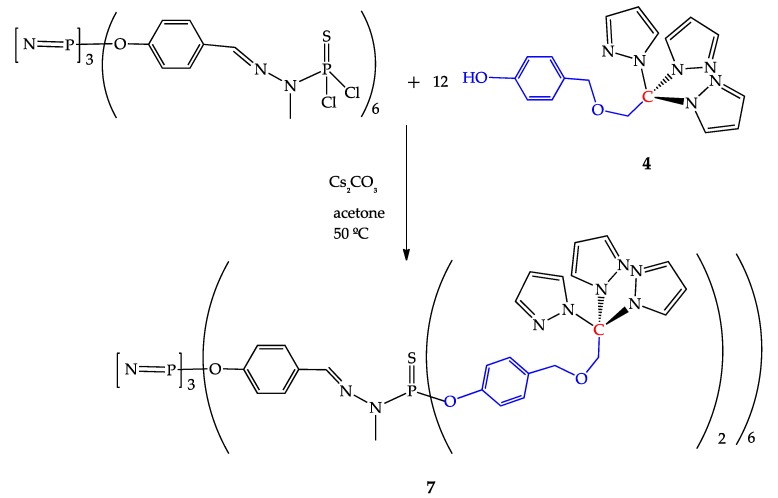
Grafting reaction of 4-(2,2,2-tris(1-pyrazolyl)ethoxymethyl)phenol (**4**) on the N_3_P_3_-G_c1_ dendrimer, affording N_3_P_3_-G_c1_-[CH_2_C(pz)_3_]_12_ (**7**).

Based on the nature of the functional groups on the dendritic terminations, tris-2,2,2-(1-pyrazolyl)ethanol (**1**) has been selected as a promising candidate for the final step of condensation with the phosphorus dendrimer N_3_P_3_-G_c1_: the hydroxy moiety of the scorpionate holds, in principle, the nucleophilic character to react with the thiophosphonyldichloride (-PSCl_2_) terminations of the first-generation dendrimer. However, partial substitution or decomposition of the dendrimer has been detected. The hydroxy group of (**1**) was not enough active to achieve the total saturation of active terminations of the macromolecule. Moreover, the sodium alkoxide derivative (**2**) led to a partial decomposition of the building block (see Scheme S6). Therefore, the phenolic scorpionate 4-(2,2,2-tris(1-pyrazolyl)ethoxymethyl)phenol (**4**) was used as depicted in Scheme 5.

**Figure 3:**
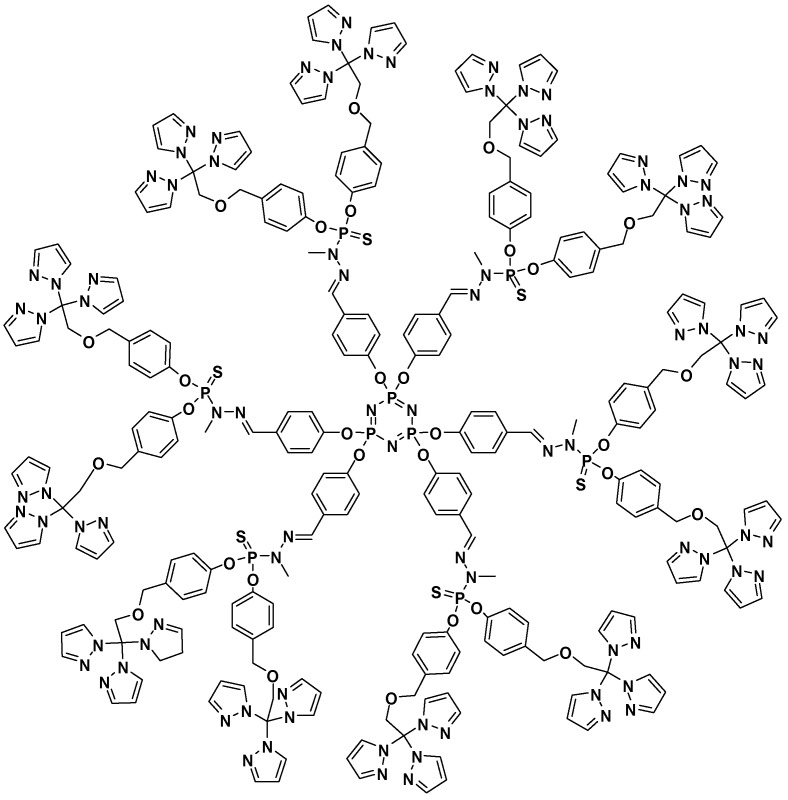
The new tris(pyrazolyl)methane-based phosphorus dendrimer N_3_P_3_-G_c1_-[CH_2_C(pz)_3_]_12_ (**7**).

The N_3_P_3_-G_c1_ dendrimer and an excess (15 eq.) of (**4**) were solubilized in acetone, 30 equivalents of caesium carbonate were added, and the mixture was left stirring for three days at 50 °C to afford (**7**) with 36 pyrazolyl functional groups as end-groups on the dendrimer (Figure 3). In the ^31^P-NMR spectra, the phosphorus signal at δ 65.85 ppm of the N_3_P_3_-G_c1_ dendrimer undergoes a shift to 65.39 ppm for the di-substituted compound (**7**). In addition, in the ^13^C-NMR spectra, the signal from the phenol group in **4** at δ 156.39 ppm is shifted to 149.68 ppm for (**7**).

**Scheme 6:**
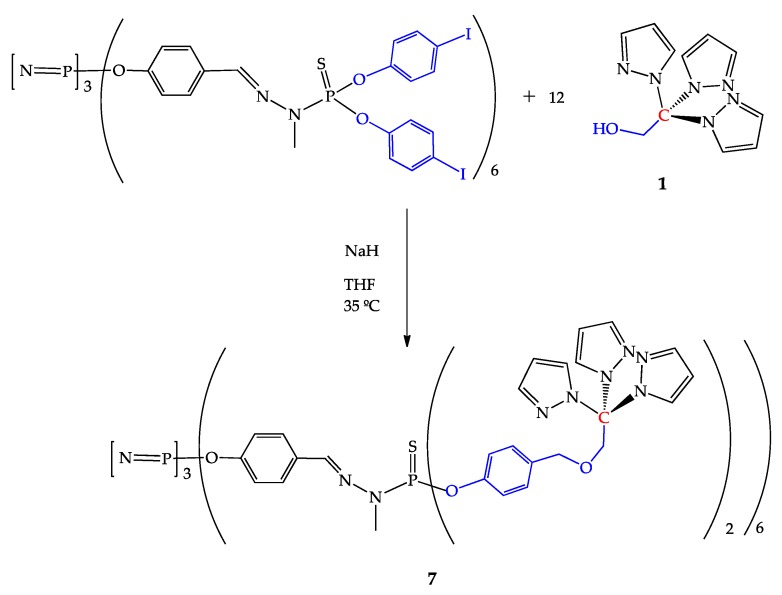
Grafting reaction of tris-2,2,2-(1-pyrazolyl)ethanol (**1**) on the N_3_P_3_-G_c1_-I_12_ dendrimer, affording N_3_P_3_-G_c1_-[CH_2_C(pz)_3_]_12_ (**7**).

A synthetic alternative using the iodo-benzyl derivative (N_3_P_3_-G_c1_-I_12_) [49] of the dendrimer molecule in the presence of tris-2,2,2-(1-pyrazolyl)ethanol (**1**) and sodium hydride was also successful to obtain the tris(pyrazolyl)methane-based phosphorus dendrimer N_3_P_3_-G_c1_-[CH_2_C(pz)_3_]_12_ (**7**) (Scheme 6) in moderate yield.

### 2.3. Catalytic Studies

In situ generated Pd complexes of tris-2,2,2-(1-pyrazolyl)ethanol (**1**) and of its new tris(pyrazolyl)methane-based phosphorus dendrimer N_3_P_3_-G_c1_-[CH_2_C(pz)_3_]_12_ (**7**) were chosen to act as catalysts for the industrially important Sonogashira and Heck C-C couplings. Nitrogen-donor ligands are known to enhance the catalytic activity of palladium(II) acetate [50,51] for such C-C bond formation processes. The in situ formation instead of using already prepared complexes was applied to simplify the process, since in most cases there is no difference in the catalytic activity between pre-formed and in situ generated catalysts [52].

**Scheme 7:**
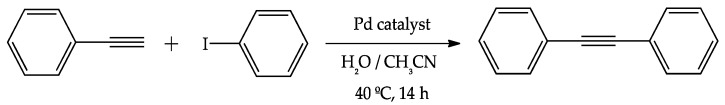
Sonogashira C-C coupling between phenylacetylene and iodobenzene catalysed by Pd-(**1**) or Pd-(**7**).

The Cu-free Sonogashira C-C coupling of phenylacetylene and iodobenzene was performed according to Scheme 7 at 40 °C (oil bath) during 14 h in the presence of Pd(OAc)_2_ and (**1**) or (**7**). Since neither of the ligands were soluble in water the experiment was started by solubilizing palladium and ligand in the acetonitrile and allow reacting for 15 min before water, both substrates and the base (triethylamine) were added.

**Figure 4:**
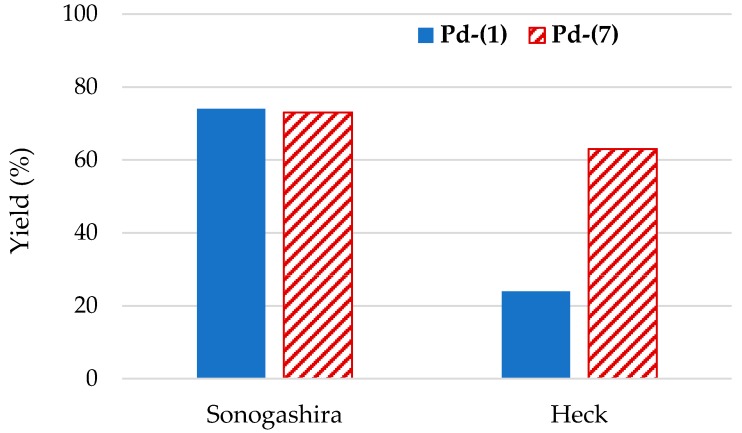
Yields obtained for Sonogashira and Heck C-C couplings catalyzed by Pd-scorpionates formed in situ.

Very similar results for both scorpionate compounds were obtained, yielding 74 and 73% of diphenylacetylene respectively for Pd-complexes (**1**) and (**7**) (see Figure 4). However, when the alcohol scorpionate (**1**) was used, a mirror of Pd(0) on the glassware was detected suggesting that the scorpionate dendrimer (**7**) leads to a more stable catalyst than does (**1**). It has been observed with phosphorus dendrimers having thiazolyl phosphine Pd-complexes as terminal functions that no Pd leaching could be measured by inductively coupled plasma mass spectrometry (ICP-MS) with the dendrimer, whereas the leaching was 1432 (±46) ppm with the corresponding monomer [53].

**Scheme 8:**
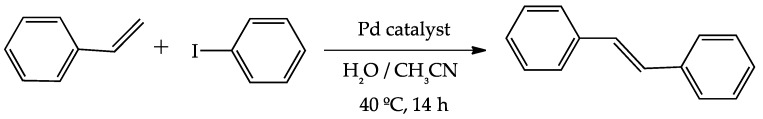
Heck C-C coupling between styrene and iodobenzene catalysed by Pd-(**1**) or Pd-(**7**).

The catalytic activity of in situ generated Pd complexes of (**1**) and (**7**) was also tested for the Heck C-C coupling of styrene and iodobenzene. The reaction was performed according to Scheme 8 at 70 °C (oil bath) during 14 h in the presence of Pd(OAc)_2_ and (**1**) or (**7**). Pd compound and the ligand were solubilized in acetonitrile and left reacting for 15 min before the water, both substrates and the base (triethylamine) were added.

In this case, the alcohol scorpionate (**1**) afforded only 24% conversion whereas the dendrimer (**7**) led to almost three times (63%) more product with excellent selectivity (Figure 4).

In all Heck experiments, the selectivity for *trans*-stilbene was 100% (the only product detected). The mirror of palladium(0) observed on the glassware when using the Pd-(**1**) catalyst was probably the reason for such a low catalytic activity.

This work revealed that the anchorage of a tris(pyrazolyl)methane derivative on a dendritic surface is feasible and generates an important support for further studies in supramolecular and coordination chemistry, and in catalysis. In fact, it allowed to extend the scope of C-C bonds formation catalyzed [54,55] by tris(pyrazolyl)methane type metal catalysts. The obtained results show that the synthetic process requires further investigation to ease the preparation of intermediates and improve yields.

## 3. Materials and Methods

All syntheses were carried out under an atmosphere of dinitrogen or Argon, using standard Schlenk techniques. All solvents were dried, degassed and distilled prior to use. The reagents (Aldrich and Acros) were purchased and used without further purification. Where indicated, the reagents were synthesized in accordance with literature methods.

C, H and N analyses were carried out by the Microanalytical Service of the Instituto Superior Técnico. Infrared spectra (4000−400 cm^−1^) were recorded on a BIO-RAD FTS 3000MX instrument (Hercules, CA, USA) in KBr pellets and far infrared spectra (400–200 cm^−1^) were recorded on a Vertex 70 spectrophotometer, in polyethylene and cesium iodide pellets. Vibrational frequencies are expressed in cm^−1^; abbreviations (intensity, shape): s, m and w, strong, medium and weak; s and br, sharp and broad. Ultra-violet, visible and near infra-red (UV/vis/NIR) spectra (1600–200 nm) were recorded on a Shimadzu UV-3101PC UV-VIS NIR spectrophotometer (Duisburg, F.R., Germany). ^1^H-, ^13^C- and ^31^P-NMR spectra were recorded with a Bruker AC 200, ARX 250, AV 300, DPX 300, AMX 400 or AV500 spectrometers (Billerica, MA, USA). ^1^H and ^13^C chemical shifts δ are expressed in ppm relative to Si(Me)_4_. The reference for ^31^P-NMR chemical shifts is 85% H_3_PO_4_. The attribution of ^13^C-NMR signals has been done using *J*mod, two-dimensional HSQC, HMBC, and HMQC, Broad Band or CW ^31^P decoupling experiments when necessary. Coupling constants are in Hz; abbreviations: s, singlet; d, doublet; m, complex multiplet; vt, virtual triplet; br, broad. Unless stated otherwise, numbering of pyrazolyl ring atoms is as depicted in Figure 1. ESI+/ESI-mass spectra were obtained on a VARIAN 500-MS LC ion trap mass spectrometer (solvents: acetonitrile/methanol; flow: 20 μL/min; needle spray voltage: ±5000 V, capillarity voltage: ± 100 V; nebulizer gas (N_2_): 35 psi; drying gas (N_2_): 10 psi; drying gas temperature (N_2_): 350 °C).

Hydrotris(pyrazolyl)methane and tris-2,2,2-(1-pyrazolyl)ethanol (**1**) were synthesized according to published procedures [32].

### 3.1. Synthesis of Odium tris-2,2,2-(pyrazol-1-yl)ethanoate, NaOCH_2_C(pz)_3_ (***2***), (pz = pyrazolyl)

Sodium hydride (75 mg, 1.88 mmol, 1 eq., 60% dispersion in mineral oil) was washed with dry pentane (2 × 15 mL) and then suspended in dry THF (15 mL). A THF (20 mL) solution of tris-2,2,2-(pyrazol-1-yl)ethanol (**1**) (456 mg, 1.87 mmol, 1 eq.) was added dropwise to the hydride mixture under dinitrogen with H_2_ evolution. Then, the residue obtained by evaporation of the solvent was crushed in dry pentane (4 mL) leading to the pale-yellow solid (**2**) in quantitative yields. ^1^H-NMR (300 MHz, CDCl_3_): δ 7.70 (d, 3H, *J* = 2.5 Hz, 3-H (pz)), 7.11 (d, 3H, *J* = 2.5 Hz, 5-H (pz)), 6.36 (dd, 3H, *J* = 2.6 Hz, 4-H (pz)), 5.08 (s, 2H, CH_2_).

### 3.2. Synthesis of (tris-2,2,2-(pyrazol-1-yl)ethoxy)benzyl, PhCH_2_OCH_2_C(pz)_3_ (***3***), (pz = pyrazolyl)

Sodium hydride (75 mg, 1.88 mmol, 1 eq., 60% dispersion in mineral oil) was washed with dry pentane (2 × 15 mL) and then suspended in dry THF (15 mL). A THF (20 mL) solution of tris-2,2,2-(pyrazol-1-yl)ethanol (**1**) (456 mg, 1.87 mmol, 1 eq.) was added dropwise to the hydride mixture under dinitrogen; during this time, gaseous H_2_ was formed. A THF (5 mL) solution of benzyl chloride (388 μL, 3.36 mmol, 1.8 eq.) was added dropwise to the final solution. The resulting pale brown solution was refluxed overnight. Then the mixture was allowed to cool down to room temperature and H_2_O (20 mL) and Et_2_O (30 mL) were added. The organic phase was separated, and the aqueous phase was washed with Et_2_O (10 mL). The organic extracts were collected, washed with brine and dried over Na_2_SO_4_, whereafter they were filtered, and the solvent removed under vacuum to leave a pale-yellow solid that was purified by column chromatography (acetone/pentane 4/6) leading to a white powder of (**3**) (41%). Compound (**3**) is well soluble in all common organic solvents Me_2_CO, CHCl_3_, CH_2_Cl_2_, MeOH, EtOH and DMSO, and no soluble in H_2_O. IR (KBr): 3101 (m s), 3022, 2951, 1534 (s s, ν (C=N)), 1519 (s s, ν (C=C)), 1413 (m s), 858 (m s), 738 (s s), 482 (m s) cm^−1^. ^1^H-NMR (300 MHz, CDCl_3_): δ 7.63 (d, 3H, *J* = 2.4 Hz, 5-H (pz)), 7.42 (d, 3H, *J* = 2.4 Hz, 3-H (pz)), 7.29 (m, 3H, m, p-H (Ph)), 7.18 (d, 2H, *J* = 6.0 Hz, o-H (Ph)), 6.31 (dd, 3H, *J* = 2.5 Hz, 4-H (pz)), 5.11 (s, 2H, CH_2_-C(pz)_3_), 4.49 (s, 2H, CH_2_-Ph).

### 3.3. Synthesis of 4-(2,2,2-tris(1-pyrazolyl)ethoxymethyl)phenol, 4-OH-C_6_H_4_CH_2_OCH_2_C(pz)_3_ (***4***) (pz = pyrazolyl)

In a round bottom flask containing 23 mg (0.81 mmol) of NaH were added 5 mL of THF (dist.). To this grey slurry was drop wise added a 20 mL THF (dist.) solution containing 0.20 g (0.81 mmol) of tris-2,2,2-(pyrazol-1-yl)ethanol (**1**) and 0.24 g (0.81 mmol) of protected4-(bromomethyl)phenol. When the drop wise addition was finished, the reaction mixture was left stirring at reflux for 17 h. A dilution was made with H_2_O followed by an extraction with 3 × 20 mL ether. The organic phases were collected and washed with saturated NHCO_3_ and brine, dried with MgSO_4_ and evaporated. Yield: 87%. The product was a pale-yellow powder (C_24_H_47_N_6_O_2_Si_1_, Mw: 479.77 g/mol). During this step a certain percentage of the protection was removed, giving a spectrum of the mixed products. As the next step was the deprotection; the product was used as such. ^1^H-NMR (200 MHz, CDCl_3_): δ 0.19 (s, C_11_H, 6H), 0.97 (s, C_13_H, 9H), 4.42 (s, C_5_H, 2H), 5.09 (s, C_6_H, 2H), 6.31–6.34 (m, C_9_H, 3H), 6.77 (d, C_2_H, ^3^*J* = 8.4 Hz, 2H), 7.05 (d, C_3_H, ^3^*J* = 8.4 Hz, 2H), 7.42 (d, C_10_H, ^3^*J* = 3.0 Hz, 3H), 7.65 (d, C_8_H, ^3^*J* = 1.7 Hz, 3 H). ^13^C-{^1^H}-NMR (63 MHz, CDCl_3_): δ -4.39 (s, C_11_), 25.69 (s, C_13_), 25.71 (s, C_8_), 30.34 (s, C_12_), 73.90 (s, C_6_), 77.59 (s, C_5_), 89.90 (s, C_7_), 106.52 (s, C_9_), 119.98 (s, C_2_), 129.33 (s, C_4_), 130.15 (s, C_3_), 131.03 (s, C_10_), 141.24 (s, C_1_), 141.33 (s, C_8_).

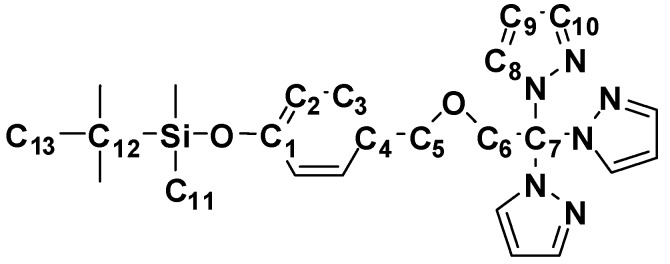



In a round bottom flask, 0.326 g (0.7 mmol) of the above compound was solubilized in 8 mL of THF. The solution was cooled to 0 °C in an ice bath and 0.443 g (1.4 mmol) of tetrabutylammonium fluoride was added with care. The ice bath was removed and left stirring until ambient temperature was reached. The mixture was diluted by 20 mL of ethyl acetate and washed with 3 × 20 mL of water. The organic phase was dried with MgSO_4_ and evaporated until dryness. For further purification a column chromatography was prepared with hexane/ethyl acetate (1:1, Rf: 0.36). Yield: 90%. The product was a pale-yellow powder (C_18_H_18_N_6_O_2_, Mw: 350.38 g/mol). ^1^H-NMR (300 MHz, CDCl_3_): δ 4.42 (s, C_5_H, 2H), 5.08 (s, C_6_H, 2H), 6.34–6.35 (m, C_9_H, 3H), 6.64 (d, C_2_H, ^3^*J* = 8.4 Hz, 2H), 7.00 (d, C_3_H, ^3^*J* = 8.4 Hz, 2H), 7.44 (d, C_10_H, ^3^*J* = 2.4 Hz, 3H), 7.68 (d, C_8_H, ^3^*J* = 1.2 Hz, 3H). ^13^C-{^1^H}-NMR (75 MHz, CDCl_3_): δ 72.77 (s, C_6_), 73.91 (s, C_5_), 89.93 (s, C_7_), 106.63 (s, C_9_), 115.42 (s, C_2_), 128.16 (s, C_4_), 129.72 (s, C_3_), 131.12 (s, C_10_), 141.44 (s, C_8_), 156.39 (s, C_1_).

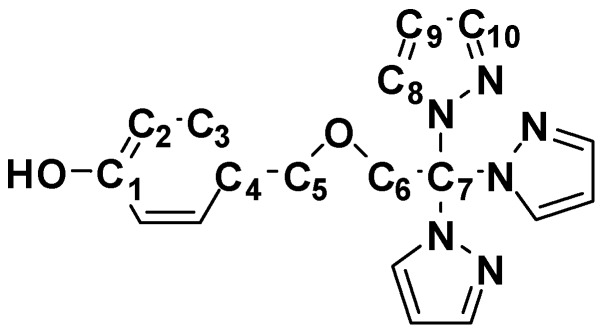



### 3.4. Synthesis of N-tosyl-2-(tris-2,2,2-(1-pyrazolyl)ethoxy)ethaneamine, TsNHCH_2_CH_2_OCH_2_C(pz)_3_ (***5***) (Ts = para-toluenesulfonyl, pz = pyrazolyl)

Sodium hydride (202 mg, 5.07 mmol, 1 eq., 60% dispersion in mineral oil) was washed with dry pentane (2 × 15 mL) and then suspended in dry THF (20 mL). A THF (20 mL) solution of tris-2,2,2-(pyrazol-1-yl)ethanol (**1**) (1.23 g, 5.07 mmol, 1 eq.) was added dropwise to the hydride mixture under dinitrogen for 20 min.; during this time, gaseous H_2_ was formed. A THF (10 mL) solution of *N*-tosylaziridine (1 g, 5.07 mmol, 1 eq.) was added dropwise to the final solution. The resulting pale-yellow solution was heated at 50 °C for 2 h. The mixture was allowed to cool down to room temperature and H_2_O (20 mL) and CHCl_3_ (30 mL) were added. The organic phase was separated, and the aqueous phase was washed with CHCl_3_ (10 mL). The organic extracts were collected, washed with brine and dried over Na_2_SO_4_, whereafter they were filtered, and the solvent removed under vacuum to leave a pale-yellow oil. Compound (**5**) was isolated by column chromatography of this oil (acetone/pentane 1/2) leading to a white powder of (**5**) (52%). Compound (**5**) is well soluble in all common organic solvents, Me_2_CO, CHCl_3_, CH_2_Cl_2_, MeOH, EtOH and DMSO, and no soluble in H_2_O. ^1^H-NMR (400 MHz, CDCl_3_): δ 7.68 (d, 3H, *J*= 2.5 Hz, 5-H (pz)), 7.56 (d, 2H, *J* = 8 Hz, m-H(Ts)), 7.14 (d, 2H, *J* = 8 Hz, o-H(Ts)), 7.04 (d, 3H, *J* = 2.5 Hz, 3-H (pz)), 6.30 (dd, 3H, *J* = 2.5 Hz, 4-H (pz)), 6.09 (tr br, 1H, *J* = 6 Hz, NH), 4.90 (s, 2H, CH_2_-C(pz)3), 3.56 (tr, 2H, *J* = 6 Hz, CH_2_-O), 2.97 (q, 2H, *J* = 6 Hz, CH_2_-NH), 2.48 (br, 3H, H_3_C(Ts)). ^13^C-NMR (400 MHz, CDCl_3_): δ 143.12 (H_3_C-C(Ts)), 141.92 (3-C(pz)), 136.87 (O_2_S-C(Ts)), 130.45 (5-C(pz)), 129.58 (m-C(Ts)), 127.06 (o-C(Ts)), 106.93 (4-C(pz)), 89.53 (C(pz)_3_), 73.78 (H_2_C-C(pz)_3_), 69.77 (CH_2_-O), 42.25 (CH_2_-NH), 21.50 (H_3_C(Ts)).

### 3.5. Synthesis of TsNHCH_2_CH_2_TsNCH_2_CH_2_OCH_2_C(pz)_3_ (***6***) (Ts = para-toluenesulfonyl, pz = pyrazolyl)

Compound (**6**) was isolated (23%) from chromatographic purification of (**5**) at the final stage. Higher yield (41%) of (**6**) was obtained by modifying the preparation of (**5**) in the following way: the solution of sodium tris-2,2,2-(pyrazol-1-yl)ethanoate was then added dropwise to a solution of N-tosylaziridine. Compound (**6**) is well soluble in all common organic solvents, Me_2_CO, CHCl_3_, CH_2_Cl_2_, MeOH, EtOH and DMSO, and no soluble in H_2_O. ^1^H-NMR (400 MHz, CDCl_3_): δ 7.65 (d, 3H, *J* = 2.5 Hz, 5-H (pz)), 7.60 (d, 2H, *J* = 8 Hz, m-H(Ts)), 7.29 (d, 2H, *J* = 8 Hz, o-H(Ts)), 7.18 (d, 3H, *J* = 2.5 Hz, 3-H (pz)), 6.32 (dd, 3H, *J* = 2.5 Hz, 4-H (pz)), 5.82 (tr br, 1H, *J* = 6 Hz, NH), 5.00 (s, 2H, CH_2_-C(pz)_3_), 3.63 (tr, 2H, *J* = 6 Hz, CH_2_-O), 3.21 (tr, 2H, *J* = 6 Hz, O-CH_2_-CH_2_-NTs), 3.21 (tr, 2H, *J* = 6 Hz, NH-CH_2_-CH_2_-NTs), 2.96 (q, 2H, *J* = 6 Hz, CH_2_-NH), 2.44 (br, 3H, H_3_C(Ts)).

### 3.6. Synthesis of the Scorpionate Dendrimer, N_3_P_3_-G_c1_-[CH_2_C(pz)_3_]_12_ (***7***)

Route 1: Sodium hydride (16 mg, 0.41 mmol, 24 eq., 60% dispersion in mineral oil) was washed with dry pentane (2 × 3 mL) and then suspended in dry THF (5 mL). A THF (5 mL) solution of tris-2,2,2-(pyrazol-1-yl)ethanol (**1**) (100 mg, 0.41 mmol, 24 eq.) was added dropwise to the hydride mixture under dinitrogen; during this time, gaseous H_2_ was formed. A THF (5 mL) solution of N_3_P_3_-G_c1_-I_12_ ([4198.38], 68 mg, 16.67 × 10^−3^ mmol, 1 eq.) [49] added dropwise to the final solution. The resulting solution was stirred at 35 °C overnight. The solvent was evaporated under vacuum and the residue was suspended in chloroform (8 mL), filtered and washed with ether (2 × 15 mL) affording a pale-yellow powder of (**7**) (32%).

Route 2: 0.134 g (0.4 mmol) of compound **4** and 0.05 g (27 µmol) of N_3_P_3_-G_c1_ dendrimer were solubilized in 1 mL of acetone. To the solution was added 0.25 g (0.8 mmol) of Cs_2_CO_3_. The reaction mixture was left stirring at 50 °C for three days. The solution was evaporated, solubilized in chloroform, filtered and washed with two portions of ether. Yield: 47%. The product was a pale-yellow powder. (**7**), C_264_H_252_N_87_O_30_S_6_P_9_, (Mw: 5594.68 gmol^−1^). ^1^H-NMR (300 MHz, CDCl_3_/THF-d_8_): δ 2.90–3.30 (br s, C_0_^6^H, 18H), 4.39 (br s, C_1_^5^H, 24H), 5.09 (s, C_1_^6^H, 24H), 6.26 (br s, C_1_^9^H, 36H), 6.60–7.25 (br m, C_0_^2^H + C_1_^2^H + C_0_^3^H + C_1_^3^H, 72H), 7.38 (br s, C_1_^10^H, 36H), 7.57 (br s, C_0_^5^H + C_1_^8^H, 42H). ^31^P-{^1^H}-NMR (121 MHz, CDCl_3_/THF-d_8_): δ 11.86 (s, P_0_), 65.39 (s, P_1_). ^13^C-{^1^H}-NMR (75 MHz, CDCl_3_/THF-d_8_), 32.36 (br s, C_0_^6^), 72.73 (s, C_1_^6^), 72.90 (s, C_1_^5^), 89.26 (s, C_1_^7^), 105.74 (s, C_1_^9^), 120.73 (s, C_0_^2^ + C_1_^2^), 127.68 (s, C_0_^3^), 128.25 (s, C_1_^3^), 129.15 (d, C_0_^5^, ^3^*J*_CP_ = 14.2 Hz), 130.26 (s, C_1_^10^), 131.78 (s, C_0_^4^), 134.03 (s, C_1_^4^), 140.45 (s, C_1_^8^), 149.68 (br s, C_1_^1^), 150.69 (br s, C_0_^1^).

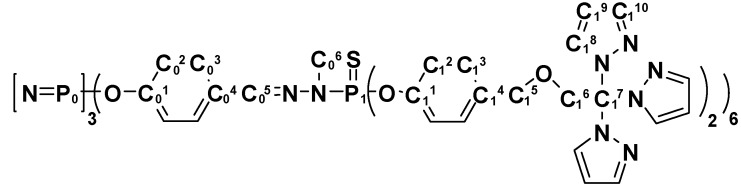



### 3.7. Catalytic tests

All catalytic reactions were performed in Schlenk tubes, with strong magnetic stirring, and warm oil bath. The percentage of conversion and the selectivity were measured by relative integration of ^1^H-NMR signals. Experiments have been done in duplicate, and the values given are the mean values (generally ±2).

**Sonogashira C-C coupling**: at 40 °C (based on oil bath) during 14 h with (phenylacetylene: iodobenzene: ligand: Pd: base) (100:110:1.1:1:120) (6.0 mg, 26.9 µmol, Pd(OAc)_2_). Since neither of the ligands were soluble in water, the experiment was started by solubilizing the palladium and ligand in the acetonitrile (6 mL) and leaving it reacting for 15 min before the water (1 mL), substrate A and B and triethylamine (the base) were added. The reaction was considered to start when the heating commenced.

**Heck C-C coupling**: We used 70 °C (oil bath) and 14 h with (styrene: iodobenzene: ligand: M: base) (100:100:1.1:1:120) (6.0 mg (26.9 µmol) Pd(OAc)_2_) and the base used was triethylamine. We solubilized the palladium and ligand in acetonitrile (6 mL) and left them reacting for 15 min before the water (1 mL), substrate A and B and the base were added. The reactions were considered to start when the heating commenced.

## Supplementary Materials

The supplementary materials are available online.
