# Supplementary material for: Novel Methinic Functionalized and Dendritic C-Scorpionates
Source: Molecules. 2018 Nov 23;23(12):3066. doi: 10.3390/molecules23123066 (PMC6321409; doi:10.3390/molecules23123066)
Supplement: Supplementary file 1 [file molecules-23-03066-s001.pdf]

Supplementary Information for

Novel methinic functionalized and dendritic C-scorpionates

Luísa M.D.R.S. Martins, [1,*] Riccardo Wanke, [1] Telma F.S. Silva,[1] Armando J.L. Pombeiro,[1] Paul Servin,[2,3] Régis Laurent,[2,3] Anne-Marie Caminade[2,3]

[1] Centro de Química Estrutural, Instituto Superior Técnico, Universidade de Lisboa, Av. Rovisco Pais, 1049–001 Lisboa, Portugal;

[2] Laboratoire de Chimie de Coordination du CNRS, 205 route de Narbonne, BP 44099, F-31077 Toulouse Cedex 4, France

[3] LCC-CNRS, Université de Toulouse, CNRS, Toulouse, France

* Correspondence: luisammartins@tecnico.ulisboa.pt; Tel.: +351-218419269

Preparation of the phenolic C-scorpionate 4-(2,2,2-tris(1-pyrazolyl)ethoxymethyl)phenol from tris-2,2,2-(1-pyrazolyl)ethanol and *tert*-butyl dimethyl silyl protected 4-(bromomethyl)phenol.

The first step was the protection of the phenol of 4-hydroxybenzaldehyde. By reacting 4-hydroxybenzaldehyde with one equivalent of tertbutyldimethylsilanechloride and one equivalent of imidazole in THF we received the *tert*-butyl dimethyl silyl protected 4-(bromomethyl)phenol (see Scheme S1). In ^{13}C NMR we saw a change of the signal of the carbon in the meta position to the phenol. It was changed down field from 116.06 ppm for 4-hydroxybenzaldehyde to 120.37 ppm for the product.

By reducing the phenol protected molecule with one equivalent of $NaBH_4$ in THF over night we managed to receive the corresponding alcohol compound (see Scheme S1) with a retained protection on the phenol function. Both in ^{13}C and in ^{1}H NMR there were large differences between the starting material and product. In ^{1}H NMR the aldehyde disappeared at 9.83 ppm and the benzyl alcohol function appeared at 4.57 ppm. In ^{13}C NMR the same difference was expected and seen. The aldehyde shifted up field from 190.58 ppm to 64.96 ppm for the benzyl alcohol.

The next step was to make a substitution reaction of the alcohol for a better leaving group. Here we might have been a bit too cautious and should probably have gone directly to the bromide compound and spared ourselves one reaction step. But we did not and instead we reacted the alcohol compound with one equivalent of tri-fluoroacetic anhydride in THF. The reaction mixture was stirred for 40 minutes at reflux to yield us the CF_3COO derivative (see Scheme S1). In ^{1}H NMR we saw the peak for the benzyl group shift from 4.57 ppm to 5.31 ppm for the product.

Another nucleophilic substitution to receive an even better leaving group was done by reacting the above compound in refluxing THF for 14 hours with one equivalent of LiBr giving us the desired *tert*-butyl dimethyl silyl protected 4-(bromomethyl)phenol (see Scheme S1). In ^{1}H NMR we once again saw a change of the benzyl group. This time it changed up field from 5.31 ppm to 4.51 ppm

which was very similar to the previous value with the alcohol function. In ^{13}C we also saw a change up field from 69.57 ppm for the benzyl group on the starting material to 33.93 ppm for the product.

Finally, we are at an important step of this synthetic route since we are about to graft the scorpionate onto the phenolic species. A slurry was made with NaH in THF. To this slurry was slowly added a mixture containing one equivalent of alcohol scorpionate and the *tert*-butyl dimethyl silyl desired protected 4-(bromomethyl)phenol. The reaction was left stirring for 17 hours before we received *tert*-butyl dimethyl silyl protected (tris-2,2,2-(pyrazol-1-yl)ethoxy)-4-phenol (see Scheme S1). In ^{1}H NMR we observed a minor change of the benzyl function from 4.51 ppm to 4.42 ppm for the product. In ^{13}C NMR the change of the benzyl function was more noteworthy since the starting material was at 33.93 ppm and in the product the material ended up at 77.59 ppm.

One tiny little step left to have the product that we started the crusade for. This step was a deprotection of the phenol function and was done by solubilizing the *tert*-butyl dimethyl silyl protected 4-(2,2,2-tris(1-pyrazolyl)ethoxymethyl)phenol in THF and cooling it in an ice-bath. To this cold mixture two equivalents of tetrabutylammonium fluoride were slowly added and was left stirring until ambient temperature was reached giving us 4-(2,2,2-tris(1-pyrazolyl)ethoxymethyl)phenol (see Scheme S1). In ^{1}H NMR the di-methyl group at 0.19 ppm as well as the tert-butyl group at 0.97 ppm from the protecting group had completely disappeared.

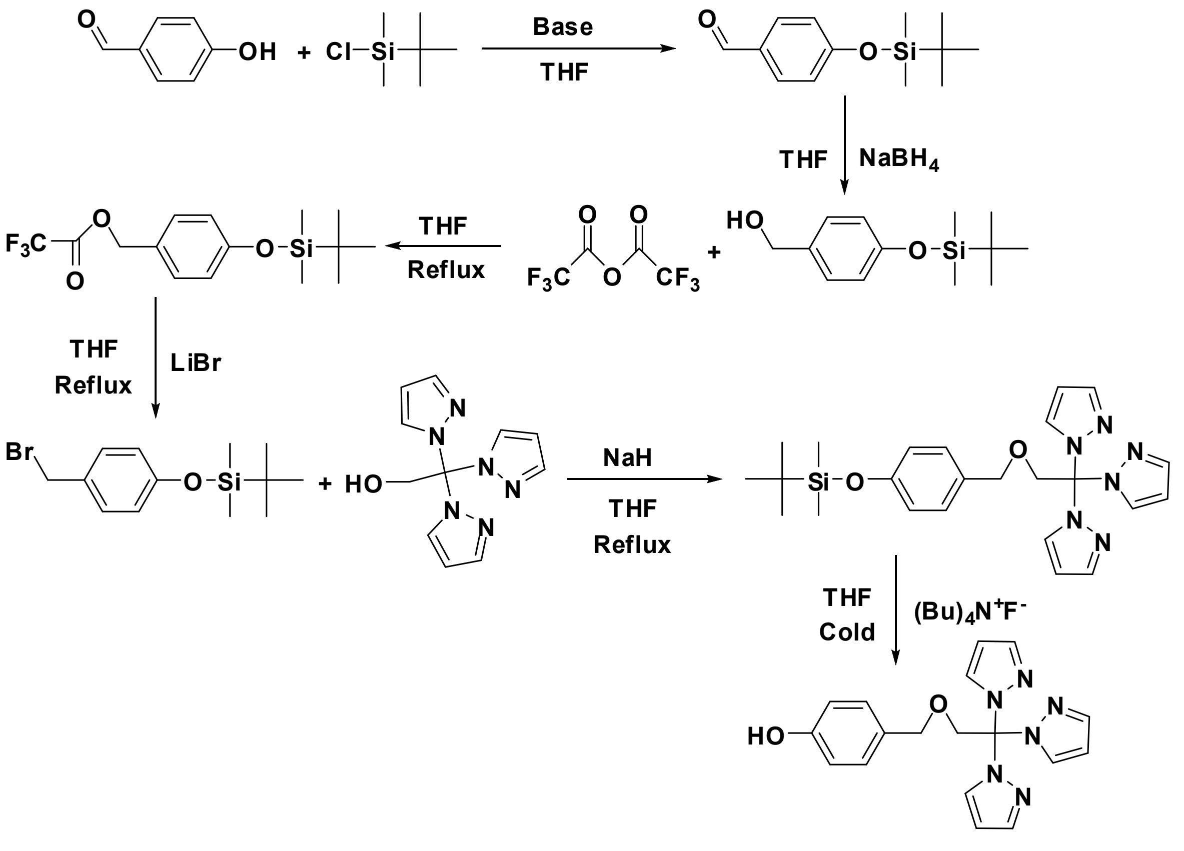

Scheme S1. *The (tedious) route to prepare the phenolic C-scorpionate (**4**).*

Preparation of *N*-tosylaziridine from ethanolamine

N-tosylaziridine was obtained by a two steps pathway:

1) Synthesis of N,O-bistosylethanolamine, TsNHCH₂CH₂OTs. To a stirred solution of tosylchloride (6.17 g, 32.36 mmol, 2.1 eq) in pyridine (4 mL) cooled at -30 ºC was added dropwise a solution of ethanolamine (993 µL, 16.18 mmol, 1 eq) in pyridine (3 mL). The resulting mixture was stirred 1 h at -10 ºC, overnight, at 0 ºC. Crushed ice and $CHCl_3$ (10 mL) were added and the mixture stirred for 15 minutes. The organic phase was separated and washed with H_2O (3 x 10 mL), glacial acetic acid (5 mL) and water (10 mL), dried over Na_2SO_4 and evaporated to yield an orange oil that was triturated in pentane affording a white off solid (81%). ^{1}H-NMR (300 MHz, $CDCl_3$): 7.75 (d, 2H, JHH = 8 Hz, m-H(Ts-O)), 7.70 (d, 2H, JHH = 8 Hz, m-H(Ts-N)), 7.36 (d, 2H, JHH = 8 Hz, o-H(Ts-O)), 7.31 (d, 2H, JHH = 8 Hz, o-H(Ts-N)), 4.87 (tr br, 1H, JHH = 6 Hz , NH), 4.05 (tr, 2H, JHH = 6 Hz , CH_2-O), 3.23 (q, 2H, JHH= 6 Hz , CH_2-NH), 2.47 (br, 3H, H_3C(Ts-O)), 2.44 (s, 3H, H_3C(Ts-N)).

2) Synthesis of N-tosylaziridine. To a toluene solution (40 mL) of *N,O*-bistosylethanolamine (4.83 g, 13.07 mmol, 1 eq) stirred vigorously, a solution of KOH (3.30 g, 58.90 mmol, 4.5 eq) in H_2O was added dropwise for 1 h. The mixture was stirred for 2 h at room temperature, then the organic layer was separated washed with H_2O (15 mL), dried over Na_2SO_4, filtered and evaporated. The crude residue was triturated in pentane (20 mL) and dried under vacuum affording a white/yellow solid (89%). ^{1}H-NMR (300 MHz, $CDCl_3$): 7.84 (d, 2H, JHH = 8 Hz, m-H(Ts)), 7.36 (d, 2H, JHH = 8 Hz, o-H(Ts)), 2.38 (m, 4H, CH_2-CH_2), 2.48 (br, 3H, H_3C(Ts)).

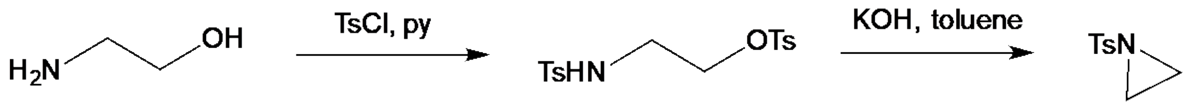

***Scheme S2.** The route to prepare tosyl-protected azidirine.*

Attempts to deprotect N-tosyl-2-(tris-2,2,2-(1-pyrazolyl)ethoxy)ethaneamine

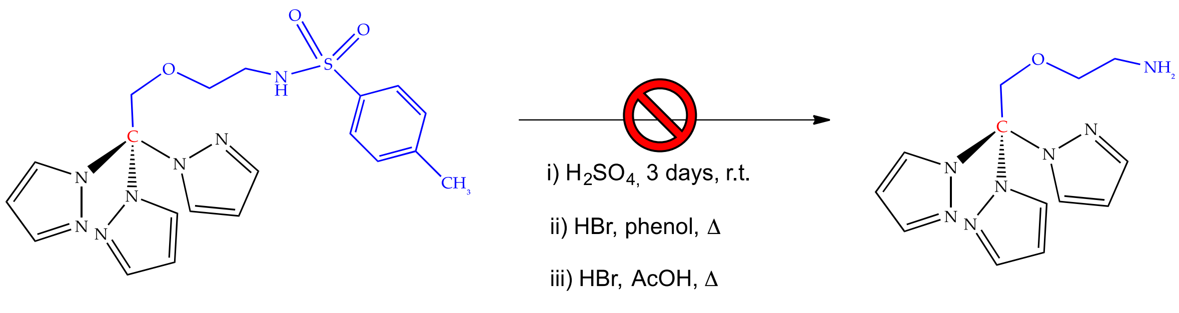

***Scheme S3.** Unsuccessful routes to prepare tosyl-protected azidirine.*

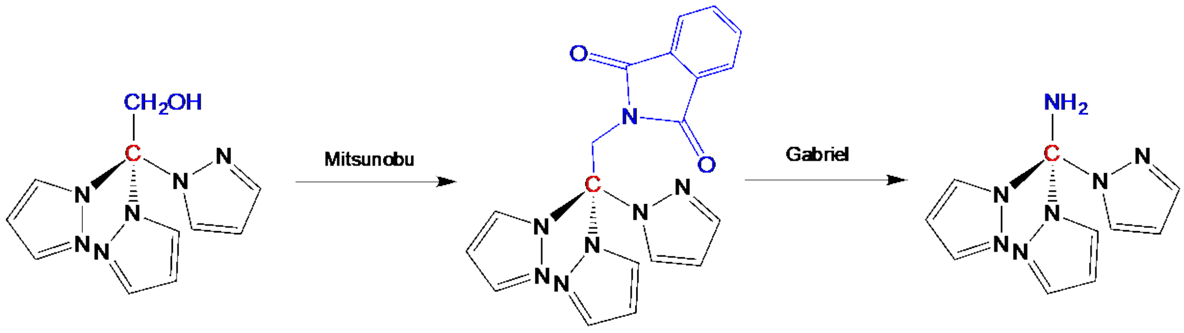

Scheme S4. *Mitsunobu/Gabriel pathway to functionalize tris-2,2,2-(1-pyrazolyl)ethanol.*

Attempts to prepare the carboxylic acid derivative of hydrotris(pyrazolyl)methane

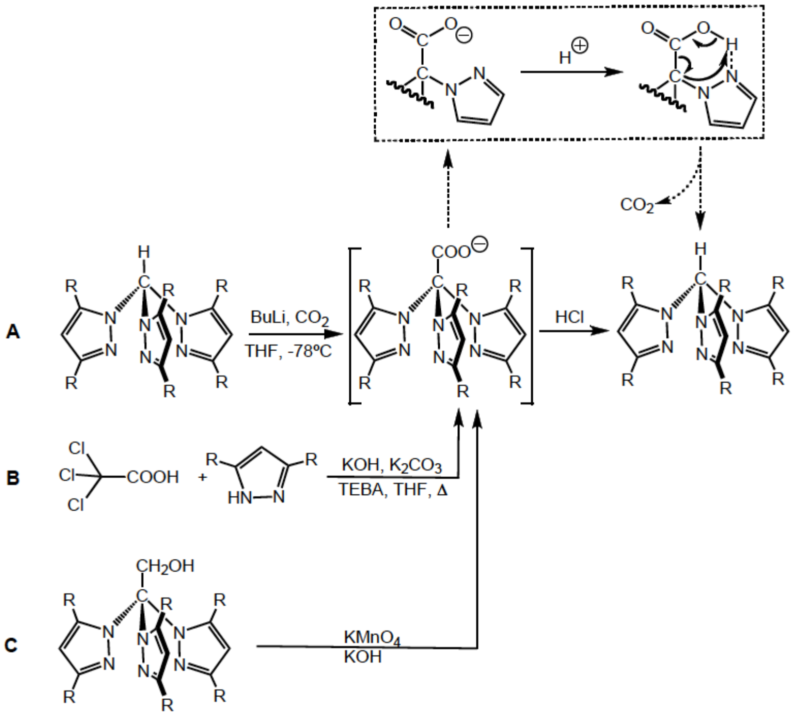

Scheme S5. *Routes for the carboxylic acid functionalization of hydrotris(pyrazolyl)methane.*

The apical proton of the variably ring substituted hydrotris(pyrazolyl)methane has been successfully removed (Reaction **A**) and the carbanion carboxylated to obtain the lithium carboxylate Li[OOC-C(pz)$_3$]. This derivative has also been achieved by nucleophilic substitution of trichloroacetic acid (reaction **B**) or by oxidation of the terminal alcohol tris-2,2,2-(1-pyrazolyl)ethanol (reaction **C**). However, at the final stage, the addition of acid to the carboxylate solution results in the formation of the starting hydrotris(pyrazolyl)methane.

A proposed decarboxylation mechanism assisted from the nitrogen atom of an adjacent pyrazolyl ring is shown in Scheme S5: the weakly basic nitrogen could promote the activation of the carboxylic group and the decarboxylation process. This decomposition mechanism from the intermediate carboxylate is suggested by the detection of the quantitative formation of

hydrotris(pyrazolyl)methane, HC(pz)$_3$, from reaction of (**2**) with KOH and $KMnO_4$ in water and subsequent acidification with HCl (reaction **C**).

In order to explore the synthetic strategies of functionalization of hydrotris(pyrazolyl)methane, a modification of a previous reaction **B** (Scheme 4) has been carried out: 3 eq of 3,5-dimethylpyrazole have been reacted with trichloro acetonitrile. The reaction does not proceed to completion but appears promising to further studies.

Unsuccessful attempts to graft tris-2,2,2-(1-pyrazolyl)ethanol or sodium tris-2,2,2-(pyrazol-1-yl)ethanoate to different first-generation dendrimers

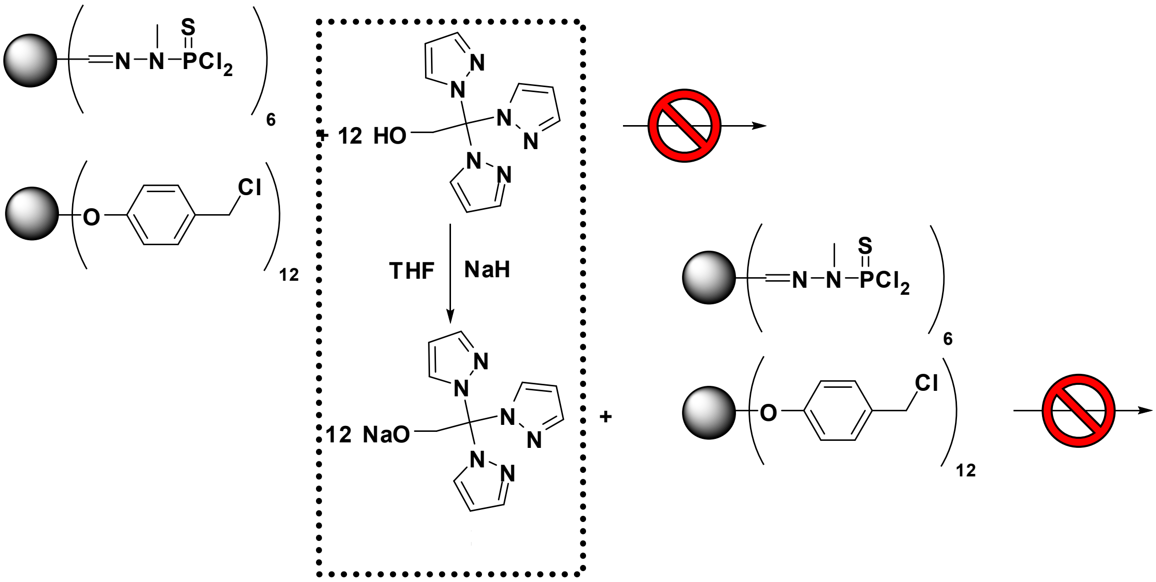

Scheme S6. *Routes for grafting tris-2,2,2-(1-pyrazolyl)ethanol or sodium tris-2,2,2-(pyrazol-1-yl)ethanoate to first generation dendrimers.*
